# Pigmented Tumour of the Eyelid with Unexpected Findings

**DOI:** 10.1155/2012/471368

**Published:** 2012-06-03

**Authors:** Anne C. Hunold, Martina C. Herwig, Frank G. Holz, Hans-Peter Fischer, Karin U. Loeffler

**Affiliations:** ^1^Department of Ophthalmology, Marienhospital Aachen, Friedrich-Ebert-Allee 98, 52066 Aachen, Germany; ^2^Department of Ophthalmology, University of Bonn, Ernst-Abbe-Straße 2, 53127 Bonn, Germany; ^3^Division of Ophthalmic Pathology, Department of Ophthalmology, University of Bonn, Ernst-Abbe-Straße 2, 53127 Bonn, Germany; ^4^University Eye Hospital Bonn, Ernst-Abbe-Straße 2, 53127 Bonn, Germany; ^5^Department of Pathology, University of Bonn, Sigmund-Freud-Straße 25, 53127 Bonn, Germany

## Abstract

A 63-year-old patient presented with a small painless nodular tumour of his left lower eyelid which had increased in size over the last few weeks. The tumour was excised by wedge resection and submitted for ophthalmopathologic examination. Histopathologic examination revealed a cystic lesion of apocrine origin with focal proliferations. The proliferative cells appeared pleomorphic and displayed marked atypia. Staining with Ki67 revealed a significant mitotic activity supporting the diagnosis of an apocrine adenocarcinoma of Moll. As the lesion displayed in most parts characteristics of a benign apocrine hidrocystoma, a thorough and critical histopathological examination is required in such cases to avoid missing an early malignant transformation.

## 1. Introduction

Apocrine adenocarcinoma is a very rare tumour that originates from apocrine glands such as the glands of Moll in the eyelid. There is no racial preference, but males are affected more often than females (5 : 3) [1]. The tumour usually presents as solitary lesion with a solid-to-cystic aspect. The most frequent location is the axilla [1–3].

Histopathologically, apocrine adenocarcinoma reveals cuboidal tumour cells with eosinophilic cytoplasm and areas of decapitation secretion [4].

The most important differential diagnoses in the eyelids are benign tumors such as apocrine hidrocystoma and cystadenoma that are frequently found in the eyelids and metastases from elsewhere in the body.

## 2. Case Presentation

**Figure 1:**
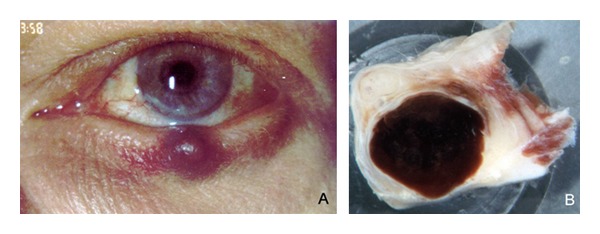
(A) Clinical photo with a well-circumscribed brownish nodule at the left lower eyelid. (B) Gross examination of the bisected tumor with a circumscribed dark cyst within the tarsus.

A 63-year-old patient was referred with a small painless nodular tumour of his left lower eyelid (Figure 1(A)). The lesion was noted about six months ago and had increased in size during the last weeks. Slit lamp microscopy revealed a lesion with a smooth surface near the lid margin. There was some pigmentation on the tarsal conjunctiva which was suspected by the referring ophthalmologist to represent a melanocytic process.

Visual acuity was 20/30 in both eyes. Apart from bilateral pseudophakia and st/p Nd:Yag capsulotomy, the previous ocular and medical history was uneventful.

Because of recent growth and unknown etiology, the tumour was excised by wedge resection and submitted for ophthalmopathologic examination. The specimen measured 8.5 mm × 8.5 mm × 5.5 mm. After bisecting the specimen, gross examination revealed a dark cystic lesion in the region of the tarsus (Figure 1(B)).

**Figure 2:**
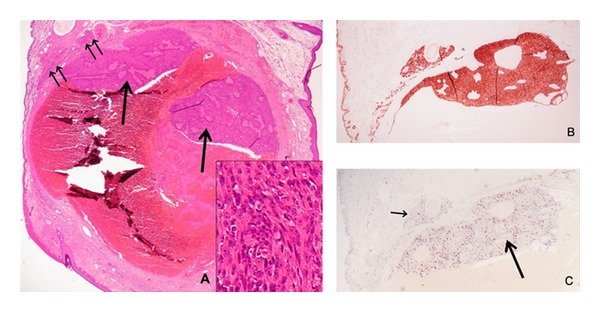
(A) Microscopic view showing a hemorrhagic cyst with proliferations into the lumen (single arrow) as well as in the adjacent tissue (double arrow) (H&E, 10x). The cells are pleomorphic and atypical (insert; H&E, 40x). (B) The tumor cells are strongly positive for CK18 indicating its “simple” epithelial nature (10x). (C) With anti-Ki67 a high proliferative activity is seen within the luminal part of the tumor (big arrow; 10x) as well as in the adjacent tumor nodule (small arrow; 10x).

Histology demonstrated a cystic lesion near the lid margin lined by a double-layered epithelium with an inner columnar layer and an outer flattened myoepithelial layer. There was an inflammatory pseudocapsule present similar to an ordinary apocrine hidrocystoma. However, deeper sections displayed focal proliferations of the lining epithelium into the lumen (Figure 2(A)). Within these proliferations, the cells were pleomorphic and revealed marked atypia with significant mitotic activity. Outside the lumen, small clusters of similar tumour cells were also detected. Close to the lid margin the cyst wall was replaced by inflammatory cells, and the adjacent fibrous tissue exhibited small haemorrhages, multiple macrophages, foreign body giant cells, and cholesterol clefts. With Prussian blue, numerous cells reacted positive for iron in this area, and immunohistochemistry revealed multiple CD68 positive cells confirming the inflammatory reaction surrounding the lesion.

All epithelial cells including those of the epidermis, the conjunctiva, and the lining of the cyst as well as the tumour cell proliferations within and outside the lumen were homogenously labelled with pancytokeratin. In contrast, CK18 (Figure 2(B)) was solely expressed by the epithelium of glandular origin including the tumour cells and to a lesser degree by the conjunctival epithelium. With Melan A, occasional melanocytes within the squamous cell epithelium were labelled but all tumour cells were negative. Ki67 was positive in about 30% of the tumour cells (Figure 2(C)) and in basal cells of the epithelium. Considering the high proliferative activity, the invasive growth pattern, and the pleomorphic appearance of the cells in some areas of the tumour, the lesion was diagnosed as apocrine adenocarcinoma arising from an apocrine hidrocystoma of a gland of Moll.

## 3. Discussion

This case describes an apocrine adenocarcinoma most probably originating from the glands of Moll at the lid margin that was mimicking an apocrine hidrocystoma.

The literature reveals only a few cases of possible adenocarcinoma originating from glands of Moll. Aurora and Luxenberg postulated three criteria to designate a tumor of the ocular adnexae to be of apocrine origin: first, the site of growth—the tumour should be located at the lid margin; second, the cells are expected to have a strongly eosinophilic cytoplasm with areas of apocrine decapitation; third, iron-positive intracellular granules in up to one-third of the tumours [3]. Our tumor met the first two of these criteria and thus fit into this category.

Differential diagnoses include mainly simple apocrine hidrocystoma, apocrine cystadenoma, and metastatic lesions. From a clinical point of view, tumours such as hemangioma, lymphangioma, atypical basal cell carcinoma, or even malignant melanoma may have a similar appearance.

Apocrine hidrocystoma often appears as solitary lesion in the face and neck area. Multiple hidrocystomas can be associated with the Schopf-Schulz-Passarge syndrome and the Gorlin-Goltz syndrome [5]. In the literature, the terms “apocrine hidrocystoma” and “apocrine cystadenoma” are often used interchangeably. Sugiyama et al. differentiated in their study apocrine lesions with papillary projections into the cystic cavity (apocrine cystadenoma) from lesions without papillary projections (apocrine hidrocystoma). Apocrine cystadenoma can be further distinguished in a proliferative group with true papillary growth and a group with pseudopapillary growth. In the proliferative group, they demonstrated a significantly higher rate of atypia, Ki67 staining, and extraluminal proliferation/infiltration. Thus, proliferative apocrine cystadenoma exhibits features similar to apocrine carcinoma, and the distinction can be rather difficult. A metastatic lesion was highly unlikely, as the tumor was rather localized, and a primary tumor did not exist.

The malignant potential of apocrine adenocarcinoma is not well established since only a few cases have been reported in the literature. Most patients seem to have a favourable outcome, although orbital and intracranial extensions as well as lymph node metastases may occur [2, 6, 7]. A few cases with aggressive behaviour of an apocrine adenocarcinoma have been described [6, 7].

In concordance with a similar case presented by Seregard [1] our patient did not suffer from any recurrence or metastatic disease during three years of followup.

##  Authors' Contribution

A. Hunold and M. Herwig contributed equally to this paper.
